# Abstract of Proceedings of the Buffalo Medical Association

**Published:** 1867-10

**Authors:** T. M. Johnson

**Affiliations:** Sec’y.


BUFFALO

### Medical and Surgical Journal.

VOL. VII. OCTOBER, 1867. No. 3.

### Original Communications.

ART. I.—*Abstract of Proceedings of the Buffalo Medical Association.*

Tuesday Evening, September 3d, 1867.

The President and Vice President being absent, Dr. S. W. Wetmore was elected Chairman of the meeting.

Members present—Drs. Wetmore, Daggett, Diehl, Kamerling, Gleason, Congar, Cronyn, Samo, Hauenstein, Gay, Abbott, Potter, Smith, Mackay and Johnson.

The minutes of the last meeting were read and approved.

Drs. Conrad Diehl, C. F. A. Nichell and Byron H. Daggett, were elected to membership.

Dr. J. J. Edmonds was proposed for membership.

The Committee on By-Laws asked for further time, which was granted.

Dissertations on designated subjects being in order, Dr. H. M. Congar read the following dissertation:

*A Section of the Etiology of Uterine Disorders.*

Mr. President and fellows of the Buffalo Medical Association:

Gentlemen:—In the course of a life of practical medicine, there is no one of us who does not arrive at conclusions in regard to some one or more of the evolutions of human nature, which are overlooked by the mass of the profession; and to which majority, consequently, such final deductions are new, strange and surprising not only, but who, should they tend to invalidate valued prejudices, respected opinions, or to curtail, or deny cherished pleasures' present or prospective, sometimes reject, oppose, and even occasionally denounce them, and especially their advocates. Notwithstanding, in our search for a theme, in compliance with your appointment for this evening, which should, at the same time, entertain, instruct and elevate, we have concluded to present you *one of these very medical uniquities*, relying upon your generosity, disinterestedness and friendliness in the charitable interpretation of our motives, in the deliberate judgment of the effects of fact and principle, and in the forbearance of our inevitable imperfections of representation.

In the second year of our practice, (1834) there were two female patients, whose peculiarities are yet distinctly remembered. They were about the same age, had been married about the same length of time, had seven or eight children apiece, and had been nearly bed-ridden for five or six years, from prolapsus uteri, purulent leucorrhœa, chronic bronchitis, and anæmia. They were tall, spare, and, in complexion, brunettes. Their husbands were blondes, tall, large framed, hard-working farmers. As often as the uterine disorders were relieved by treatment, the activity and intensity of the bronchitis without treatment, also abated; and whenever they were aggravated the severity of the pulmonary affection was proportionately increased. When the secondary character of this bronchitis was, to our mind, clearly established, we looked about for other cases. Nor did we long look in vain, for we found them scattered freely throughout that whole region. These cases of secondary pulmonary affections furnished the data for our thesis for graduation in the winter of 1836–7, at Geneva Medical College, having practiced upon county license during the preceding five years. These two husbands died while their wives were in this bed-ridden state. During the first five years of their *widowhood* they gradually, steadily and permanently so improved without any medical treatment as to be free from every symptom of uterine and pulmonary disorders, and continued so when last heard from a few years ago. About three years after leaving that region,

(1840) two other female patients came under our medical attention with the same kind of uterine disorders, just widowed, and each at her father's house in the country. Each without any special uterine medical treatment, in about seven years' widowhood, recovered health completely, re-married, the one having subsequently one, and the other six sons, without any marked return of the uterine affections, which at the close of their first marriage proved very nearly fatal.

During the last twenty-five years cases like these four have been continually appearing to our medical vision, the description of which we spare you. Sir, it is in vain for us to disguise the fact, that this *widowhood cure* of inflammation, ulceration and hypertrophy of the uterus (for when first noticing these facts, specular treatment of uterine affections was unknown in America) made a deep and lasting impression upon us; and led to the notice of another large class of cases of the same affections, which were so improved by the *absence of the husband from home*, some to Europe, a few to Asia, many to the Pacific States and territories, and others to various other sections of our extensive domain, an *absence* varying from three to thirty months, an *improvement* so generally *proportioned to the chronological rest* of the uterine functions, as to merit the cognomen of the *absence cure*, the description of which we also spare you. And no one will be surprised that this last class of cases so deepened the impression made by the first, as to cause us to inquire, could *excess* in the *use of matrimonial privileges* have produced these primary uterine, and these secondary pulmonary disorders, which were so unmistakably relieved and *cured by entire rest of uterine functions?*

In 1833 the works of J. H. Bennett "*On the Uterus*," concentrated our attention to the *answer* of the above question, in presenting us with a graphic description of uterine disorders, so worded and arranged as to show a most siriking *similarity* in local and general symptoms and course of evolution, to the progress and symptomatology of the non-specific masculine disorders of the sexual apparatus from venereal excess; and enabled us to give to ourselves a most decisive affirmative. We shall endeavor briefly to exhibit *this similarity*. In paralleling these male and female affections, we shall take the disorders of the masculine apparatus as the standard of comparison. We find in the human male that the effects of venereal excesses in the healthy do not at once show themselves, but are manifested by a process of gradual evolution; for, it is only after some little time has elapsed that even a state of irritation is first noticed. This is manifested by *heat* in the urethra, when micturating, which soon extends to the bladder, as evidenced by an increased frequency of micturation, and then to the kidneys, as proved by the pains in the loins, and the increased quantity of urine passed in the twenty-four hours. The heat in the urethra is next increased to a tickling, not always disagreeable and a painful itching; the meatus of the urethra is more red and deeply injected; ejaculation is somewhat more rapidly accomplished, and with a slight diminution of the usual pleasure. The state of irritation extends and intensifies. It reaches the prostrate and margin of the anus, as evidenced by the sense of weight and uneasiness in the rectum and perineum, and by the rigidness of the sphincter ani; the intensity of coitive pleasure now rapily diminishes; ejaculations become very rapid; this irritation follows the cords to the testicles, both of which become very sensitive to, and very painful upon, pressure. About this time, in some, dysuria, and in others occasionally, hematuria, come on, with more or less blood in the semen. Now, in proportion to the diminution of the pleasure of the act, the erections become less prolonged, less complete, and the ejaculations more rapid: so that, when the pleasure is nearly extinct, the ejaculations become so precipitous that intromission cannot take place; in fact, the duration of the act, and of all the functional phenomena, is so reduced to a mere point of time, as to consist simply of the discharge of the semen. Now the emission occurs without emotion, without pleasure, without dreams, and even without any particular sensation whatever; in this condition the patients are not aware when emissions have taken place, except by the stain on their linen when they awake; in this state also the testicles are found soft and reduced, and the cords are relaxed and varicose.

Just following the affections of the prostrate, etc., as above stated, this state of irritation will be found to have passed into subacute, or chronic inflammation; it is sometimes found in the *urethra*, or in the *seminal vesicles*, or in the *vasa defferentia*, or in the *testicles*, or in the *bladder*, or in even the *kidney itself*, or in some *two* or *more of these organs contemporaneously*.

We will now turn to the local symptoms of like origin in the female, symptoms of what are called *uterine affections*. Here, too, excesses do not at once produce disorder. The first, that is noticed, is heat in the course of the vagina, followed by an increase of its transparent mucus, of its natural secretion, succeeded by a white semi-opaque secretion. This is accompanied by more or less tenderness of the parietes of the vagina; slight swelling, which is soon followed by pruritus of the vulva. This swelling and pruritus, when followed by tenderness of the vulva is always accompanied with, and is therefore a sure sign of inflammation of the os tincæ. The vulvar irritation soon extends to the urethra, causing a sense of heat in micturition, then to the bladder, producing frequent micturition, then to the kidneys, causing pain in the lumbar regions, and an increased quantity of urine. In the meantime, the white vaginal discharge soon becomes yellow, giving evidence of the secretion of pus from excoriated mucous surfaces; after a short time, in the vaginal discharges, there is noticed a transparent adhesive matter, which also after a while becomes purulent, proving thereby the passage of the inflammation and ulceration from the orifice to the cavity of the neck of the uterus. From the moment of this extension of the morbid affection, there has been a growing sense of weight and bearing down in the pelvis, and an increasing dysmenorrhœa. The pelvic weight and bearing down, and dysmenorrhœa, are sooner or later followed, when not accompanied by dull, deep-seated pains in the lumbo-sacral, ovarian, and hypogastric regions, which after a while become constant; by which it is manifest that the state of irritation has passed from the cavity of the neck to that of the body of the uterus, thence through the fallopian tubes to the ovaries. This uneasiness in the ovaries often amounts to the pain and other symptoms of ovaritis. From the time of the existence of disease in the uterus through the progress of its extension to the ovaries, there has been a gradual decrease of pleasure in the functional excitement of the sexual organs, succeeded by indifference, and terminating in disgust and loathing of it by both the married and the unmarried. Now here we have a complete parallel of disorders in their course and progress, by which organ after organ is attacked until the whole apparata are unfitted for the normal functions for which they were created, in the male terminating in impotence, in the female ending in sterility, and in both closing with an extreme aversion to amorousness, and a disgust of each other.

We now turn to the general symptoms, commencing as above with the male; pallidity, lassitude, greater in the morning than in the evening; debility, which is increasing; skin dry, but palms of the hands moist; physical development retarded, and its progress slow; coldness in the extremities; an increasing sensitiveness to cold, heat and changes of temperature; frequent sighing; the gaze mostly averted, and not the expression of a modest confidence when fixed on you; palms of the hands now clammily moist; heaviness and oppression in the head; giddiness; noise in the ears; irritability of temper; sleep broken and unrefreshing; restlessness; timidity; frightful dreams; sadness; dislike of society; melancholy; cornea dull; countenance expressionless; impaired memory; inability for concentration of thought, and other kinds of mental exertion; changes of the character and often entire loss of the voice; wandering pains over any and every part of the body; rheumatism; irregular and constant involuntary movements of the extremities; unsteadiness of gait in walking; paralysis; palpitations; shortness of breath; sense of suffocation, increased by exertion; edema of the lower extremities; hatred of existence; tenderness of the epigastrium; tongue red at its tip and edges; appetite capricious, or entire anorexia; digestion of animal food difficult, and that of vegetables producing uneasiness, flatus and flushing of the face; vomiting every now and then after taking food; abdomen distended with flatus; constipation alternating with diarrhœa; colic, sometimes very severe; marasmus, and death.

We will now look at the secondary affections in the female. Countenance pale in blondes, and sallow in brunettes; skin under the eyes slightly bluish, or leaden colored; the breasts often become large, swollen, tender, and painful; headache, especially frontal more or less intense; lassitude, especially in the morning; development of body more or less retarded; rotundity of figure lost; often imperfect vision and audition; sleeplessness; disagreeable dreams; the morning languor increasing to a very significant debility; sterility; general depression; marked timidity; great change and irritability of temper; great melancholy; emaciation increasing; the skin flaccid and dry, and the palms of the hands clammily moist; the muscles flabby; urine loaded with lithates; great sensitiveness to cold, heat, and changes of temperature; the extremities cold; the pulse generally becomes small, slow, and weak; increasing inability to exercise care, attention to the duties, and forethought concerning domestic and family interests; tongue red at the tips and edges; capricious appetite; flatulence; constipation; anorexia; frequent nausea, and pain upon the ingestion of food; foul tongue; palpitation; flushing of the face; sense of suffocation upon all quick movements; extreme debility; exhaustion, and death. Here again is a parallelism—a parallelism of general symptoms, progressively evolved from the digestive, nutritive, circulatory, motary and nervous apparata! Now it is an axiom of reason, "that like causes produce like effects." But we have presented you with similar and parallel effects. Is it not legitimate to infer, even in the absence of all other proof, that these two sets of parallel local and general symptoms, have a like or identical causation? If, however, to our inference, that *a large proportion of uterine disorders originate in matrimonial sexual excess*, we now add the evidence of this very conclusion, which was given by our *widowhood and absence cures*, are we not justified in believing and asserting that inference and conclusion to be *fact* in a certain proportion of cases? We think so.

And just here, does not the question spontaneously arise to your minds, what is excess of sexual intercourse in matrimony? If most married women have, to a greater or less extent, uterine disorders, and a large proportion of these uterine disorders originate in sexual excess, *what amount of coition is not excess in matrimony?* We shall now proceed very briefly to answer this question. If we look to the opinions of the learned for our answer, we find no two that agree, except those as in the case of the Germans, who follow the leadings of their great men, as they do of J. D. Michaels, who regarded one weekly season of amorous fruition the *rule*, and more frequently *not much out of the way*, while the mass of humanity catch greedily at all that can be obtained! But, do you ask, is there no standard in relation to this matter? We unhesitatingly answer, yes! But it is *where* you would, at first thought, least expect to find it! It is in the Bible! *A book, in the original languages of which, whose every letter, every word, every clause, every sentence, every paragraph, every book, and whose whole is authentic and inspired truth!* In this book our God and Savior has, in the chastest and most delicate phraseology, given us just such a standard, a set of laws in relation to the subject, complete, the most of them having a purely physiological basis; and yet, given to humanity, hundreds of years before there was in existence any such thing as a proper, and written medical, and, especially any physiological science.* Therefore we shall use the few scripture quotations here adduced, *as matters of undisputed fact, event and principle.*

* If objection is made to the precedent of introducing into scientific disquisitions supports and proofs from Sacred Scripture, (as has been done) that in effect it manifests a weak cause, is a desecration of the sacred volume, and is therefore disrespectful to an erudite audience, on

We first inquire, how should men and women enter into their first marriages? And answer, by quoting Gen. iii, 7: "And the eyes of both of them were opened, and they saw that they were naked; and they sewed fig-leaves together, and made themselves aprons." Now, if this first human pair, as husband and wife, had, before the fall in Paradise, amorous desires without a higher inciting and governing motive than mere pleasure, and that higher motive, in turn, unincited and uncontrolled by God himself,* that is, had irregular desires, and gratified them unreservedly like the moderns, as Milton supposed when he wrote in verse 740, of his "Paradise Lost," viz:

"This said unanimous, and other rites
Observing none, but adoration pure,
Which God likes best, into their inmost bower
Handed they went; and eas'd the putting off
Those troublesome disguises which we wear,
*Side by side were laid; nor turn'd, I ween,*
*Adam from his fair spouse; nor Eve the rites*
*Mysterious of connubial love refused;*"

what could now have made them ashamed to be in each other's sight? The simple facts were, perfectly self-governed, and awaiting the signal from God, they had as yet had no sexual connection at all; but, at once, after their disobedience, the power of self-government having been broken by the severance of their union to God, the sight of the personal charms of each aroused irregular and unbidden amorous desire in the other. This instantly alarmed them; and, in accordance with the moral instinct of self-preserva-

---

whose generous forbearance such authors arrogantly venture, we answer: Every man of thought will agree with us; 1st, that the Holy Scriptures should never be quoted in reference to any subject which is to be examined exclusively or mainly by *disputation*, because opposing parties are placed in the false position of antagonism to matters revealed by divine inspiration; 2d, that they should not be irreverently, unnecessarily, frivilously quoted, [and surely none can truthfully say, that this has been done in our paper]; but, 3d, that while being appreciated as made, given, and revealed for *human benefit*, [and not man for theirs,] they may, with all propriety and reason, be ever used in the same manner, and for the same or a like purpose as that to which they were originally adapted, and actually applied by the people to whom they first came, because God, human nature, and truth, never change; therefore, for such objects, we suppose the *anthropological facts*, *the physiological principles*, *and the hygienic laws* of revelation to be, at least, just as good, and their relevant and judicious *employment* just as *creditable* to an author and to his productions, as like facts, principles and laws, which have been evolved from French, English, or American science.

* Mark, we do not say, *amorous "feeling" incited and governed by a higher motive, and that higher motive in turn controlled by God himself*, for this is just what we are confident this first pair did possess! Consequently, to infer from what we have in our paper said, that, "*in order to have the amorousness necessary to propagate his species, man must fall*," *is illegitimate, illogical, and irrational!* We will in this connection drop a word in relation to the procreation of progeny before the fall. The animal world improves only upon trials, and those of the hardest kinds! Man improves only upon *tests of his virtue*, and those of the severest characters! Should we therefore be surprised, when reason suggests, that, probably God did not allow the first human pair to beget offspring until after their first trial, in order that, if successful in passing through it safely and profitably, their children might be daguereotypes of their excellencies not only, but of the improvement of this trial; and if unsuccessful, of the demerit and deterioration of their failure, therein rising and falling together?

tion, they promptly sought to hide the parts which had thus stimulated this irregular, and therefore ruinous soul-action. Therefore, up to the time of disobedience, Adam and Eve were both virgins.

Also, Gen. iv, 1: "And Adam knew Eve, his wife, and she conceived, and bare Cain." Now, if, even after the fall, this first married couple partook amorous fruition upon every movement of desire, as do the married in modern times, would there have been any propriety in *naming specially*, as here, the *conceiving* or *fruitful season*, without, or apart from, the others? In fact, could it have been so known as to have been thus truthfully designated? For ourselves we have long since given a promptly negative answer. We couclude, therefore, that this is the *first amorous fruition of humanity!* And also that, *at its occurrence, Adam and Eve were, both of them, virgins!* Again, Gen. xxix, 21: "And Jacob said to Laban, give me my wife, for my days are fulfilled, that I may go in unto her;" and Gen. xix, 3: "Reuben, thou art *my first born, my might, and the beginning of my strength, the excellency of dignity*, and *the excellency of power.*" Jacob was evidently a virgin when he was married, when he begat Reuben, his first born, having never destroyed his *vigor*, in either *fornication*, i. e. *masturbation*, or *sexual intercourse.* In their first marriages, *men* should always in this sense be *virgins!*

And further, Deut. xxii, 13—21: "If any man take a wife, and go in unto her, and hate her, and give occasion of speech against her, and bring up an evil name upon her, and say, I took this woman, and when I came to her I found her not a maid, then shall the father of the damsel and her mother take and bring forth the evidences of the damsel's virginity to the elders of the city in the gate, and the damsel's father shall say to the elders, I gave my daughter unto this man to wife, and he hates her; and behold, he has given occasions of speech against her, saying, I found not thy daughter a maid; and yet, these are the evidences of my daughter's virginity! And they shall spread the cloth before the elders of the city, and the elders of that city shall take that man and chastise him; and they shall amerce him in a hundred shekels of silver, and give them to the father of the damsel, because he has brought up an evil name upon a *virgin* of Israel. He may not put her away all his days; but if this thing be true, and the evidences of virginity be not found for the damsel, then they shall bring out the damsel to the door of her father's house, and the men of her city shall stone her with stones that she die, because she has wrought folly in Israel to play the harlot in her father's house. So shalt thou put away *evil* from among you."

The importance of *virginity* in the first marriages of women is here distinctly asserted, even to the extreme of the death penalty for its proved absence. The reasons are briefly these: *Woman is alone adulterable.* The girl who allows herself *amours* before marriage, has hereby rendered herself incapable of bearing a pure progeny to any other man than her seducer. Her offspring by any other man will ever be hybridous; will never be accurate likenesses, never daguerrotypical images of a present husband in either character or organization. In allowing such defilement she has forever destroyed all her present husband's prospects of leaving behind him a pure and improving posterity through her, which shall be a glory to his name. She does more than murder the offspring which she may have by him; for in transmitting the lack of moral principle which allowed, and the passionate impurity which caused the defilement, she has made them innately *a means of deterioration, a curse* to the race, even for generations after her death. These *facts* cause her to be also the greatest possible disgrace to parents, brothers, sisters, and the whole circle of kindred. Therefore, *women should always in their first marriages be virgins!* Consequently the first law of matrimonial amours is, *parties of both sexes, entering first marriages, should invariably be virgins!*

In reference to the second law, we quote again Gen. 4: 1. xxv, 17: "And Adam knew Eve, his wife, and she conceived and bare Cain, and said, I have gotten a man from the Lord, *(Jahweh.)* And Adam knew his wife again, and she bare a son, and called his name Seth, for God said, she has appointed me another seed, instead of Abel, whom Cain slew. And Cain knew his wife, and she conceived and bare Enoch." Gen. vi, 4: "And also, afterward, when the *sons of God* [the spirit renewed] came in unto the *daughters of men,* [the unrenewed in spirit] and they bare children to them, these [children] became the mighty men, who were of old the men of renown." Now, when, in the procreation of Cain, of

Seth, of Enoch, and of the mighty, the renowned men of those primitive ages, the *act itself* is *designated*, the *specification* forbids the idea of a customary repetition of it, so frequent, or at such seasons, as to throw the slightest shade of doubt over a certainty of the time of, or of obscurity over a minute knowledge of all the circumstances attending the origin of the new being; for, reference is always made more or less fully to these influential adjuncts of the important event. This certain knowledge of the *time* of conception could have been consciously perceived only by woman, the wife and mother; and the matrons of this age not only, but those of the next dispensation were even more discerning than this; for Job said (Job iii, 3:) "Oh, that the night might perish, when it was said, *there has been a man-child conceived;*" and therein showed that the *time* of conception not only, but even the *sex* of the product of conception, was often, if not generally, known at the close of the coitive act by the wife. Therefore, it is certain that, with such habits and kinds of knowledge, for copulation there would never be, on the part of the wife, an earnest wish, but because of a pre-existing desire for the new being, which the *amour* was certain to produce. This desire for offspring was undoubtedly ever the *human* starting-point of the whole series of events, to which reference is here made in the above quotations. But in reality, this series has a starting-point above the human, which is shown by the fact that Eve obtained Cain as, and *knew him to be a gift*, and received Seth as, and *knew him to be a substitute seed* for Abel, *from God.* This knowledge would naturally be a consequence of a previous *assurance* of what was to come. And this assurance would be an expected result of only an earnest request, a fervent prayer for each of these special gifts, and with the assurance, a divine permission for the procreating act at such a time, in such a place, and with described surroundings, would be sure to be gained. The chronological order and succession of this series of events are perfectly natural, viz: 1st, the desire for offspring; 2d, the invocation to God for it; 3d, the favorable and permissive answer; 4th, the implicit trust in the assurance vouchsafed from God; 5th, the amorous fruition, only however, as a means of accomplishing this object, *permitted and limited by the woman herself;* 6th, the conception; and 7th, the child-birth.—

Therefore, it is manifest, that *the procurement of offspring was, in these primitive ages, the only approvable incitement to amorous fruition;* and that when approved and thus permitted by God, *it was a season to be noted for remembrance*, as here. And we find this very order and succession of this series of events accurately described, as transpiring twenty-seven hundred and fifty years after Adam, in the case of Elkanah and Hannah, in reference to the conception and birth of Samuel, the prophet. Hannah, the childless wife of Elkanah, at the time of making her offering for menstrual purification at Shiloh, when longing for offspring, prayed, wept, and there received *assurance* from God of a favorable response; and, *after a season of worship*, returned home trusting in the divine assurance, and *then* the record declares, "Elkanah knew Hannah, his wife, and the Lord (Jahweh) remembered her, (and) she bare a son, and called his name Samuel." Therefore, even at this time, among the best of the race, *desire for offspring* was the only approvable motive for indulging in matrimonial amours. And still again the wise son of Sirach, at about the two hundredth year before Christ, wrote for the use of his scholars, "Desire not a woman for pleasure." Here, then, is the second law of matrimonial amours: *after marriage the only legitimate incitement to matrimonial amours is desire for offspring, under divine guidance.*

The third law is evolved from Lev. xv, 16—18: "If any man's seed of copulation go out from him, he shall wash all his flesh in water and be unclean until the evening; and every garment and every skin on which is the seed of copulation, shall be washed with water, and be unclean until the evening; the woman also with whom man shall lie with seed of copulation, they shall both bathe themselves in water and be unclean until the evening." From the first part of this passage we understand that seminal emissions from amorous dreams and other causes, render one unfit during a limited period for unrestricted sociality, and (as we shall hereafter see) the divine services of public worship. The inconveniences of the morning and evening bathing, of the seclusion for a whole day from society and public worship, of the necessary disclosure, in the case of minors of the event to parents, and of the inevitable recognition of the cause of the social absence by their chums, without oral declaration, constituted restraints upon lascivious imaginings, and actual lechery of every kind, of the most effective character, and thereby greatly promoted chastity, continence, marriage, fruitfulness, and a healthy and disciplinable offspring; and from the last part of the quotation we perceive a provision against the depravation of the natural secretions of the sexual organs into sources of virulent and devastating diseases, which matrimonial negligence and uncleanliness would inevitably induce. The salutary restraints which the morning and evening ablutions of the whole person, the washings, both clothing and bedding of married couples after each amorous fruition, put upon the frequency of these nuptials pleasures; and the delicate manner in which the attention of the married was, by these checks, constantly directed to the *objects of coition*, viz: the production of new and improved human beings, incite by their wisdom, our admiration. Therefore, we conclude, that the third law of matrimonial amours is, The most thorough bathing of the whole person, and a full change of night apparel and body linen, should invariably and at once follow every seminal emission, nocturnal and diurnal, and every season of amorous fruition.

The fourth law is evolved from Ex. xix, 10, 11, 14, 15: "And the Lord (Jahweh) said to Moses, go to the people, sanctify them to-day and to-morrow, and let them wash their clothes, and be ready against the third day; for on the third day the Lord (Jaweh) will come down in the sight of all the people upon Mount Sinai. And Moses went down from the mount to the people, and sanctified them, and they washed their clothes; and he said to the people, be ready against the third day! *Come not at your wives!*" And also from 1st Sam. xxi, 4, 5: "And the priest answered David, There is no common bread under my hand; but there is hallowed bread, if the young men have kept themselves from women. And David answered the priest, a truth women have been kept from us about these three days, and the vessels of the young men are holy." From the first of these two passages, we learn that there should be at least a three days' interval between matrimonial amours, and engagement in public divine services; and from the second, that there should be at least a three days' interval between a season of amorous fruition and use of consecrated food. Is the principle of this law transferable and binding upon all the *spirit-*

*renewed* of this dispensation in reference to the use of the elements of the Lord's supper, as Adam Clarke and others suppose? We, for ourselves, believe, and for about twenty years have practiced it; because, in the first place, amorous fruition is, to human nature in its present condition, incompatible with the skillful, vigorous and comprehensive intellection, which is ever implied in all right divine worship; and in the second place, it is to us incompatible also with the self-denial, the self-government and the discipline of the spirit, which is indispensable to a true and full adoration of God. Therefore we conclude the fourth law of matrimonial amours to be, *No sexual intercourse is allowable for three days, at least, prior to all important and characteristic Christian public services.*

The fifth law is evolved from Lev. xviii, 19: "Thou shalt not approach to a woman to uncover her nakedness, so long as she is put apart for her uncleanness," and from Lev. xx, 18: "And the man who shall lie with a woman having the menses, and shall uncover her nakedness, has uncovered *(to destroy)* the fountain of her fertility; and she also has uncovered (*to the causes of destruction*) the fountain of her blood; *(which is the evidence of fertility;)* and they, both of them, shall be cut off from among their people." And from Lev. xv, 24: "And if any man (that is, any husband,) shall lie with her *(who has the menses)* at all, and her flowers (that is, her menstrual discharges) be (by accident) upon him, he shall be unclean seven days, and all the bed on which he shall lie shall be unclean." All that is necessary to say of these passages is, that coition during the menstrual periods, knowing them to be such, is forbidden upon the death penalty. It is clearly a physiological law—a physiological law given seven hundred and fifty years at least before the rise of any medical science, properly so-called, and uttered with the reason for its injunction declared plainly in the original Hebrew, viz: the certainty that its customary violation will inevitably produce *uterine disorder and sterility!* We therefore infer that the fifth law of matrimonial amours is, No amorous fruition is allowable during the menstrual periods.

The sixth law is evolved from Lev. xv, 2, 7, 11, 13: "When any man has a running issue out of his flesh, because of his issue, he is unclean; and he that touches the flesh of him that hath the issue, shall wash his clothes, and bathe himself in water, and be unclean until the evening; and whomsoever he that has the issue may touch, and he has not rinsed his hands in water, shall wash his clothes and bathe himself in water, and be unclean until the evening; and when he that has an issue becomes clean of his issue, he shall then number to himself seven days for his cleansing, and wash his clothes and bathe his flesh in running water, and then shall he be clean." And from Lev. xv, 19, 15, 28: "If a woman have an issue, and the issue in her flesh be blood, she shall be put apart seven days; or if she have an issue of her blood many days out of the time of her separation; or if it run beyond the time of her separation, all the days of the issue of her uncleanness shall be as the days of her separation; she shall be unclean; but when she shall be cleansed of her issue, she shall number to herself seven days, and after that she shall be clean." In the first four verses here quoted, the non-specific gonorrhœal patient was required to continue seven days continent, separate from society, from public gatherings, and from consecrated food, after the entire cessation of the discharge, like he did when the discharge was running. This requirement of continence is based, as every medical man understands, upon the liability of the organic excitement of coition, during this seven days, to cause a return of the urethral inflammation and discharge. In the last three verses here quoted, the menstrual patient was also required to continue seven days continent, separate from society, from public gatherings, and from consecrated food, after the entire cessation of the menstrual discharge, as when the menses was flowing. Every medical mind perceives this requisition of continence to be based upon the great liability of the female sexual apparatus to take on disordered action from the excitement of coition during this interdicted seven days after each menses. But do any of you say, we have been taught and have believed, that woman was most likely to conceive during this very forbidden period of the first seven days after each menses? We are not surprised, and have anticipated this very objection from the history of physiological opinions; and yet deny that the statement is fact; and bring to the support of our denial the observations of the most thorough, and, so far as we know, the most recent physiological microscopists. "In the human female," says Bischoff, "the passage of the ovum from the ovary to the uterus, occupies eight or ten days;" and he infers that sexual connection, in order to be fruitful, must take place within eight or twelve days from the cessation of the menses; and Pouchet maintains that "the ovum is fecundated *only* in the uterus, or in the lowest part of the fallopian tubes;" for, according to his statement, "the seminal fluid never penetrates so far as the ovary, and seldom, if ever, extends beyond the middle of the fallopian tubes." [Supplement to Müller's Physiology of Generation. By Baly, pp. 58, 59, 60.] Consequently, if these observations are reliable and truthful, the conceptive period of the human female is the first few days *after* the seven days, which is here by Moses interdicted to sexual intercourse; and we have here a striking illustration of the fact, which is so often perceived by the pious, among men of learning and science, that *true science and revelation always exactly coincide.* Therefore, we conclude, that the sixth law of matrimonial amours is, *no amorous fruition is allowable during seven days succeeding the cessation of the menses.*

The seventh law is evolved from cases of disease, samples of which we proceed to detail very briefly to you. Mrs. L. H. aborted in 1831, in the fourth month of her first; in 1832 she miscarried in the fifth month of her second, and in 1833, in the last of the sixth month of the third pregnancy. From the statements of this woman and her husband in relation to their matrimonial habits, we, as well as they, became convinced that amorous indulgence after conception was the *main*, if not the only cause of these matrimonial misfortunes. Again, in February, 1841, we were hurriedly called to see Mrs. P. who was dangerously flooding from her ninth abortive pregnancy in its fourth month, having never had a living child. From the statements of the husband in relation to his marital demands upon his wife, we became fully satisfied that *coition after conception* was the whole and only cause of all these numerous and successive abortions. While his sense of his wife's danger was fresh, having noticed in him a strong desire for children, we, upon the basis of it, obtained from Mr. P. a pledge of continence after his wife's next known conception. We, at this time, however, said nothing to his wife on this subject. We did not expect he would either tell his wife, or keep his pledge; and therefore were not surprised to be, in about six months, again hurriedly called to save this woman's life from her most alarmingly abortive flooding, in the same time of this her tenth pregnancy. On her convalescence, we mentioned this subject to *herself*, who, finding there was hope of going the full time, and then of having a living child by mere *abstinence from matrimonial amours*, after knowing herself to be pregnant, and by the observance of the usual hygienic measures during the month of habitual abortion, pledged to us to follow the directions given; *and she did it too.* She passed safely the full term of her next, the eleventh pregnancy, with only a few threatenings of abortion during the heretofore fatal fourth month, all of which were silenced by quiet, free bowels, and a light unstimulating diet; and in July, 1843, Mrs. P. gave birth safely to a perfectly formed, well nourished son, to the joy of the whole family circle. These cases must suffice for the present to show the fact that sometimes, at least, coition in pregnancy, causes abortion and miscarriage.

Still further, Miss Mary N., at the age of twenty-six became the second wife of Mr. O. M., when he was thirty-three years of age. They had been married about a year when he came to us asking our medical attention to his wife, who, in the sixth month of pregnancy was very ill, and he feared, going as did his first wife, who, after two miscarriages, purulent lucorrhœa, and prolapsus uteri, went into decline, and died in the fourth year of their marriage; and now, he continued, Mary has the same purulent lucorrhœa, pain in the back, side, and lower limbs, with a constant and distressing bearing down. On visiting her, although she would submit to no vaginal examination, to no special treatment, and not even to vaginal injections by herself, we were convinced of the existence of inflammation, ulceration, and hypertrophy of the mouth and neck of the uterus. On seeing her husband the next day, and informing him of the result, he asked, what *can* be done? We answered, have your wives been generous to you in the allowance of matrimonial privileges after becoming pregnant? O, yes, he replied, although my first wife, in the last year of her life, appeared to dread somewhat my embraces, because of the pain and uneasiness which she said accompanied and remained many hours after them; and so also, he continued, after a moment's pause, does Mary; and, after a moment, he added, why, doctor, what *can* be the *cause* of this trouble to *both* my wives? We responded, and you have never had a suspicion that their dutiful submission to your desires was undermining their health, that their generous deference to your pleasures was sapping the very foundation of their lives? Not the slightest, he replied, do *you* think so? We answered, we have no doubt of it. Well, it shall be so no more, was the prompt rejoinder. That, we added, is all, perhaps, and certainly the *least* that you *can* do. But mark, Mary will have a tedious and agonizing labor, and, in the first week of her lying-in, will most likely have child-bed fever, which *may* carry her off, and will certainly leave her an invalid for a long time; and you must not attribute these results of existing disease to lack of skill on our part. Well, doctor, do your best. As we expected, her accouchment was tedious and most agonizing; and on the third day afterward she was, as we feared, attacked with *puerperal metritis*, from which she was only just saved. Three years from that time she died of tuberculosis.

Mrs. C. W., primapara, would not take an anæsthetic, was in labor three and a half days, vagina so *sensitive* and *tender* as not to allow a digital examination until just previously to the close of the first stage of the labor; and was also attacked with puerperal metritis on the third day of her lying-in, which was with great difficulty subdued. We, at the time of the labor, pledged this couple that we would inform them of what were the causes of this tedious and agonizing labor, if the wife would let us know as soon as she supposed herself again pregnant. This occurred when her first child was about fifteen months old. After stating to her husband what we supposed the particular cause was, he answered, When my wife became pregnant with her first child, I was informed by unprofessional parties, but by those who, I supposed, knew all about such matters, that the more sexual intercourse the married had during pregnancy, the more relaxed the parts would become, and therefore the more easy would be the labor; consequently I exerted myself greatly to do my whole duty, and the more strenuously as the time of labor approached. He pledged us to be *continent now; and he was so.* This, her second labor, was only three and a half hours long, and in the language of the patient herself, "*appeared more like having a passage from the bowels merely, than like her first labor, and this way of having children is no trouble at all.*" This woman has since had five other children, making in all seven; and each of these five subsequent labors has been equally, or nearly as easy as the second. The lesson from these cases cannot be mistaken. The agony in labor, the puerperal metritis, and the subsequent invalidism, had, we are certain, no other causation than *coition during pregnancy.* Therefore, believing that matrimonial amours between conception and labor, when carried to any considerable extent, are invariably injurious, producing in many cases *abortions* and *miscarriages*, and in others *agonizing labor*, and *dangerous metritis* soon after lying in, we announce the seventh law of matrimonial amours, viz: *No sexual intercourse is allowable during the entire period of pregnancy.*

The eighth law is evolved from Lev. xii, 24: "If a woman shall have conceived seed, and born a male child, she shall be unclean seven days; according to the days of the separation of her infirmity shall she be unclean; and she shall continue in the blood of her purifying thirty-three days; she shall touch no hallowed thing, nor come into the sanctuary, until the days of her purifying be fulfilled." Here is the same physiological principle in action as in the sixth law; the danger of the occurrence of uterine disorder from the incited orgasm of matrimonial amours during the six weeks after the birth of sons; hence the eighth law of matrimonial amours; *No amorous fruition is allowable during the first thirty-three days after the cessation of all lochial discharges which follow the birth of sons.*

The ninth law is evolved from Lev. xii, 5: "If a woman shall have conceived seed, and borne a female child, she shall be unclean two weeks, as in her separation, and shall continue in the blood of her purifying sixty-six days." Why is the time of the lochial discharges, and of the liability to uterine disorders, here doubled respectively? We, for ourselves, answer: for about twenty years we have observed in the women we have professionally attended, that the time of lochial discharges is, at least doubled after the birth of daughters, and that they do not bear suckling them as well as they do their sons; that they are invariably more feeble. And this, our observation, accords with the observation of Prof. Martegoute, on breeding the Dishley Manchamp merino sheep, who says, "Our monthly weighings show that the ewes, which produce female lambs, are on an average of weight superior to those that produce males; and they evidently lose more in weight than these last, during the suckling period, while the ewes, that produce males, weigh less, *and do not lose as much as the others.*" [Goodale's Breeding of Domestic Animals, p. 92.] That is, mothers are in better condition when they *conceive* daughters, and are in better condition when they *wean* sons than daughters. Hence comes the ninth law of matrimonial amours, viz: *no amorous fruition is allowable during the first sixty-six days after the cessation of all lochial discharges which follow the birth of daughters.*

The tenth and last law is evolved from 1st Cor. vii, 3—5: "Let the husband render to the wife her due; and in like manner the wife also to the husband. The wife has no power over her own body, but the husband; and in like manner the husband has not also power over his own body, but the wife. Defraud not the one the other, except it be with consent for a time, that ye may give yourselves fasting and prayer; and come together again that Satan may not tempt you on account of your (tendency to) incontinency." We here say, matrimonial duty may be demanded of the husband by the wife, and of the wife by the husband, at any time from the seventh day after the cessation of the menstrual and leucorrhœal discharges, and from the thirty-third or sixty-sixth day after the cessation of all puerperal discharges, to the occurrence of conception, or to the manifestation of an approaching menses, as inclination may dictate, when not interdicted by the occurrence of incompatible religious services, as the euchrist, etc., above referred to. Therefore the tenth law of matrimonial amours is, *The husband and the wife may demand of each other amorous duty and privilege whenever sanctioned by the second, and not interdicted by the fourth, fifth, sixth, seventh, eighth and ninth of these laws.*

To recapitulate, we have found ten laws which primitively governed matrimonial amours, viz:

1—Parties of both sexes, on entering first marriages, should invariably be virgins.

2—After marriage, the only legitimate incitement to matrimonial amours, is desire for offspring, under divine guidance.

3—The most thorough bathing of the whole persons, and a full change of night apparel and body linen should invariably and at once follow every seminal emission, nocturnal and diurnal, and every season of amorous fruition.

4—The social intercourse is allowable for three days, at least, prior to all important and characteristic Christian public services.

5—No amorous fruition is allowable during the menstrual periods.

6—No amorous fruition is allowable during the seven days succeeding the cessation of each mense.

7—No sexual intercourse is allowable during the entire period of pregnancy.

8—No amorous fruition is allowed during the first thirty-three days after the cessation of all lochial discharges which follow the birth of sons.

9—No amorous fruition is allowable during the first sixty-six days after the cessation of all lochial discharges which follow the birth of daughters.

10th—The husband and the wife may demand of each other amorous duty and privilege, whenever sanctioned by the second, and not interdicted by the fourth, fifth, sixth, seventh, eighth and ninth of these laws.

We therefore conclude, in answer to the question, What is excessive coition in matrimony? That the more frequent copulation than that which the human female requires for securing to herself conception, that all coition during the menstrual periods, during the first seven days after the cessation of each menses during the whole period of pregnancy, and during the first thirty-three or sixty-six days after the entire cessation of all lochial discharges, *is excessive coition;* and, in answer to the question, What amount of coition in matrimony is not excessive? That *the varying requisitions of unperverted feminine nature for a self-assured conception can never be excess.**

And now, sir, in conclusion, if certain intractable and otherwise incurable cases of uterine disorders are relieved and cured by merely *entire rest of sexual functions* sufficiently prolonged; if the inference be entirely sound, that from the complete parallelism of the symptomatology and course of spermatorrhœa and uterine disorders, a common and identical causation is shown; in other words that uterine disorder is to the female what spermatorrhœa is to the male; if, in the primitive ante-Mosaic ages, desire for offspring, guarded by physiologic moral instincts under divine guidance, were the only approvable incitement to amorous fruition; and if, in post-Mosaic times, this motive and these instinctive guards were for public obedience expressed in detailed revelation, as quoted for the evolution of the above ten laws, and were designed still further to disclose clearly to us the amount of amorous excess which

* We had supposed before reading our paper, that the above mere recapitulation of these ten laws answered with all reasonable accurateness the two questions to which reference is made above; but inasmuch as some gentlemen present seem to think they had not been answered at all, we have added the above in parenthesis. Gentlemen, have you *there* the answers sufficiently definite?

at this time exists everywhere; and to show to us the required also amount of normal restraint upon the hitherto undisputed matrimonial privileges of the present day, we infer and believe our conclusion to be legitimate, logical, and rational, that a large majority of the uterine disorders of married life originates in (the above well-defined) irregular and excessive sexual intercourse.

And be assured, Sir, we have cheerfully complied with the requisition of this hour, and while we acknowledge on account of the great sensitiveness of all minds in regard to this special subject, to have adventurously made it the opportunity of directing the attention of the medical profession to this inadequately appreciated department of the etiology of uterine disorders, we are grateful for the thoughtful attention with which this imperfect discussion has been received by the men of science before us.

Dr. Cronyn made a few remarks in relation to the merits of the above paper.

Dr. Gay moved a vote of thanks for the paper. The motion was carried.

Dr. Gay read the following:

64 Madison Avenue,
New York, August 29, 1867.

Dear Doctor:—In my opinion your views in relation to the treatment of fractures near the joints, is essentially correct. I do not think you was fully understood, or perhaps I ought to say, you did not state your views quite so clearly as you might have done. If you mean to say that splints are too much used in their cases by many surgeons, I fully agree with you. In a pretty large proportion of their cases the fragments support themselves, or if they do not, splints do not help us. In a large proportion of cases the displacement of the fragments is of less consequence than the anchylosis which may result. In old persons, especially, is anchylosis most liable to result from long continuance of the splints. I do not think I ever kept splints on, in case of fracture near a joint, forty days—seldom thirty days—and generally not more than twenty-one days. In most cases I direct that the splints shall be removed and passive motion employed daily, as soon as the acute inflammation has subsided—generally as early as the 14th day, (I speak now of fractures near joints,) and then I lay aside the splints as soon as I can safely.

I am sincerely glad you have spoken upon this matter; for whether I have made myself understood or not, I have intended to teach that splints have a very limited use in fractures about joints. Perhaps what I have written in relation to fractures near the elbow and wrist joints, and of the patella, convey my meaning more fully than what I have written about fractures near the ankle joint. Very truly yours,

FRANK H. HAMILTON.

To Dr. Gay.

Reports on prevailing diseases being in order, all present coincided in the opinion that there was no special disease prevailing.

Adjourned. T. M. JOHNSON, M. D., Sec'y.

